# Effects over time of two platelet gel supernatants on growth factor, cytokine and hyaluronan concentrations in normal synovial membrane explants challenged with lipopolysaccharide

**DOI:** 10.1186/s12891-015-0605-3

**Published:** 2015-06-20

**Authors:** Diana L. Ríos, Catalina López, María E. Álvarez, Ismael J. Samudio, Jorge U. Carmona

**Affiliations:** 1 Grupo de Investigación Terapia Regenerativa, Departamento de Salud Animal, Universidad de Caldas, Calle 65 No 26-10, Manizales, Colombia; 2 The Terry Fox Laboratory, BC Cancer Research Centre, 675 West 10th Avenue, Vancouver, British Columbia V5Z 1L3 Canada

## Abstract

**Background:**

Platelet-rich plasma (PRP) preparations are a common treatment in osteoarthritis (OA) and inflammatory synovitis. However, there is ambiguity regarding the ideal concentration of leukocytes and platelets in these preparations necessary to induce an adequate anti-inflammatory and anabolic response in joint tissues, such as the synovial membrane. This research aimed to study, in normal synovial membrane explants (SME) challenged with lipopolysaccharide (LPS), the temporal effects (at 48 and 96h) of leukocyte- and platelet-rich gel (L-PRG) and pure platelet-rich gel (P-PRG) supernatants on the production and degradation of platelet associated growth factors (GF) (platelet derived GF isoform BB (PDGF-BB) and transforming growth factor beta-1 (TGF-β_1_)), pro-inflammatory (tumour necrosis factor alpha (TNF-α)) and anti-inflammatory cytokines (interleukin 4 (IL-4) and IL-1 receptor antagonists (IL-1ra)) and hyaluronan (HA).

**Methods:**

Synovial membrane explants (SMEs) from 6 horses were challenged with LPS and cultured for 96h with L-PRG and P-PRG supernatants at concentrations of 25 and 50 %, respectively. The SME culture medium was changed every 48h and used for determination by ELISA of PDGF-BB, TGF-β_1_, TNF-α, IL-4, IL-1ra and HA. These molecules were also determined in synovial fluid from the horses.

**Results:**

Both the 25 and 50 % PRG supernatants produced a molecular profile in the culture media unlike that of the SME challenged with LPS only. They presented GF, cytokine and HA concentrations very near to the concentrations of these molecules in normal synovial fluid when compared with the SME control groups (either with LPS or without LPS). However, in comparison with the rest of the SME treated groups, the 25 % L-PRG produced the most IL-1ra, and the 50 % P-PRG induced the sustained production of IL-4 and HA.

**Conclusions:**

These *in vitro* findings suggest that anabolic and anti-inflammatory joint responses depend on the leukocyte and platelet concentration of the PRP preparation and on the volume of this substance injected. Moreover, it is possible, that leukoreduced PRP preparations are more effective for the medical treatment of patients with OA and inflammatory synovitis.

## Background

Synovitis is a common clinical and pathological alteration observed in people with OA [1]. The clinical condition of osteoarthritic patients may be more severe when synovitis is present [2]. Osteoarthritic patients who present with mild to severe synovitis are likely to be treated using total arthroplasty surgery [2]. The main goal of OA treatment is to establish an early diagnosis, to correct the initiating causes and to commence either intra-articular, systemic or both pharmacological treatments in order to diminish pain, avoid further joint damage, and facilitate the recovery of normal joint functions [1, 3].

Platelet-rich plasma (PRP) has emerged as an important ‘regenerative’ therapy in human [4–7] and animal patients [8–10] with joint disease. Several *in vitro* [11–15], animal *in vivo* [16–18] and clinical trials support the use of PRP in OA [19]. However, the lab protocols used for both small-scale PRP preparations and larger commercial PRP preparations can yield end products with different cellular and protein (GF) and cytokine concentrations [20]. Due to such variations, these preparations yield variable results when used under clinical conditions [5, 14, 21–23].

In general, PRP used for intra-articular injection is classified into two groups [23]: leukoconcentrated PRP (L-PRP) and leukoreduced PRP (also termed pure PRP (P-PRP)). L-PRP preparations show both increased platelet (PLT) (three-five-fold or more) and leukocyte (WBC) (threefold to fivefold or more) counts with respect to basal cell counts in whole blood. P-PRP products exhibit PLT counts ranging from physiologically low to twofold and WBC counts from negligible to twofold WBC with respect to basal cell counts in whole blood [22]. Moreover, when both PRP preparations are activated with either calcium salts or thrombin, they are transformed in a platelet-rich gel (PRG) from L-PRP to L-PRG and from P-PRP to P-PRG [23].

Recent *in vitro* evidence suggests that P-PRP/P-PRG could be more suitable for tendon [24, 25] and joint treatment [11, 14, 26] than L-PRP/L-PRG because the lower PLT and WBC concentrations in these preparations induce less tissue catabolism/inflammation and more tissue anabolism than do PLT- and WBC-rich preparations [11, 14, 26]. We compare the temporal effects (at 48 and 96h) of two concentrations (25 and 50 %) of L-PRG and P-PRG supernatants with normal synovial membrane explants (SME) challenged with lipopolysaccharide (LPS). For comparison purposes, we describe the production and degradation of platelet-associated GF (platelet-derived GF isoform BB (PDGF-BB) and transforming GF beta-1 (TGF-β_1_)), pro-inflammatory (tumour necrosis factor alpha (TNF-α)) and anti-inflammatory cytokines (interleukin (IL) 4 (IL-4), and IL-1 receptor antagonist (IL-1ra)), and hyaluronan (HA) production. Further, we perform a correlation analysis between the variables studied.

There are conflictive results regarding which PRP preparation is ideal for the treatment of OA [27]. Thus, this research is designed to study the *in vitro* response of LPS-challenged SME to several PRP preparations. We investigate the hypothesis that both platelet gel supernatants at different concentrations should produce different growth factor, cytokine and HA concentrations with respect to normal synovial membrane explants and those cultured with LPS. We also explore whether the 50 % P-PRG supernatant has a better anti-inflammatory/anabolic release profile than other platelet preparations do in SME challenged with LPS.

## Methods

This study was approved by the committee on animal experimentation of the Universidad de Caldas, Manizales, Colombia.

### Samples

Synovial membrane samples from the dorsal metacarpophalangeal joints from 6 horses, 4 to 9 years of age, were included in this study. The samples were taken from horses that were free from musculoskeletal disease and euthanized by a pentobarbital intravenous overdose for other medical reasons. All metacarpophalangeal joints were radiographed and macroscopically evaluated to exclude horses with OA-associated joint changes. In addition, 2 mL of synovial fluid were obtained from each joint in order to determine the actual concentrations of PDGF-BB, TGF-β_1_, TNF-α, IL-4, IL-1ra and HA.

### Platelet concentrates (L-PRP and P-PRP) preparation

**Fig. 1:**
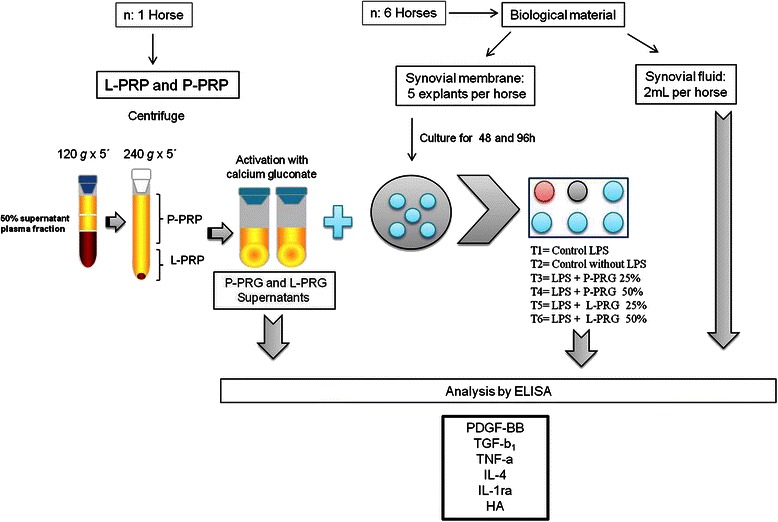
Schematic workflow of the experiments of the study

Venous blood from 1 clinically healthy 11-year-old mare was used in order to avoid great variability in the GF, cytokine and HA concentrations in the PRG supernatants used in the experiments. Platelet concentrates were obtained through a manual double centrifugation tube method [28], that was previously validated and used clinically in horses with OA [8]. Blood was drawn from jugular venipuncture and immediately deposited in 4.5 mL tubes with sodium citrate solution (BD Vacutainer®, Becton Drive, Franklin Lakes, NJ, USA). After centrifugation at 120 *g* for five minutes, the first 50 % of the top supernatant plasma fraction, adjacent to the buffy coat, was collected. This fraction was centrifuged at 240 *g* for five minutes, and then the bottom fourth of the fraction was collected [29]. This fraction was considered as L-PRP. The upper plasma fraction was considered as P-PRP (Fig. 1). Whole blood and both PRP preparations were analysed for PLT, WBC and red blood cell (RBC) concentration using an impedance-based haematology device (Celltac-α MEK 6450, Nihon Kodhen, Japan).

Both PRP preparations were activated with calcium gluconate (Ropsohn Therapeutics Ltda®, Bogotá, Colombia) (ratio 1:10) and kept in incubation at 37 °C for 1h until clot retraction occurred. Fresh L-PRG and P-PRG supernatants were used every time culture media were changed. Aliquots of both PRG supernatants obtained at every time point were frozen at -86 °C for later quantification of PDGF-BB, TGF-β_1_, TNF-α, IL-4, IL-1ra and HA.

### Synovial membrane explants culture and LPS challenge

Synovial membrane samples were obtained aseptically, and circular 4 mm diameter explants were obtained using a disposable biopsy punch (KAI Medical, Solingen, Germany). SME were dissected from the joint capsule and washed in phosphate-buffered saline. A total of 30 SME were obtained from each horse.

SME were stabilized in Dulbecco’s Modified Eagle Medium (DMEM) (high glucose, 4500 mg/L) with L-glutamine and sodium bicarbonate and free of sodium pyruvate (DMEM, Lonza Group Ltd, Basel, Switzerland) and supplemented with streptomycin (100 μg/mL) and penicillin (100 μg /mL) without the addition of serum. Cultures were incubated in a 5 % CO2 and water saturated atmosphere for 24h and then replaced with fresh culture media. At this time point, part of each tissue sample was challenged with 100 ng/mL of LPS (Sigma-Aldrich, St Louis, MO, USA) to induce inflammatory/catabolic damage of the SME [30].

### Study design

The design of the study included the evaluation of 6 experimental groups, as follows: 2 SME control groups (1 with LPS added and 1 without LPS) without addition of any PRG supernatant and 4 SME groups cultured with L-PRG and P-PRG supernatants at 2 different concentrations (25 and 50 %).

After 1h of incubation, L-PRG and P-PRG supernatants were added in order to obtain concentrations at 25 and 50 %. All SME groups were cultured at 48h, after which the culture media changed and replaced with fresh culture media and fresh PRG supernatants. The SME groups were then incubated for an additional 48h. Culture media obtained at 48 and 96h were aliquoted and frozen at -86 °C for later determination of PDGF-BB, TGF-β_1_, TNF-α, IL-4, IL-1ra and HA. The schematic diagram presented is in Fig. 1 summarizes the study’s design and methodology.

### ELISA analysis

**Fig. 2:**
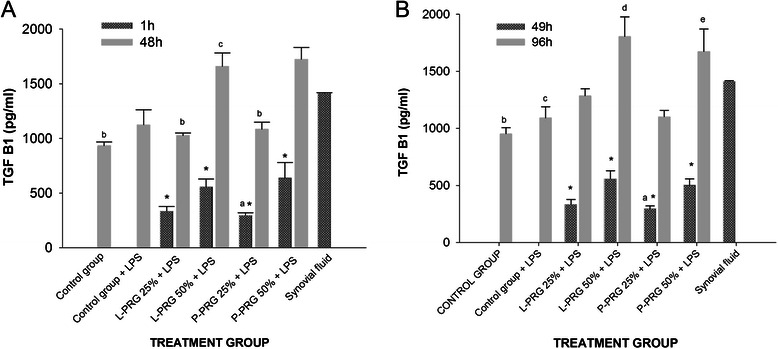
TGF-β_1_ concentrations obtained in culture media of the synovial membrane explant groups at 48 (**a**) and 96 h (**b**). ^a-b^ Lowercase letters denote significant (p < 0.01) differences between groups in the same column by Tukey test. **a** Significantly different with a: P-PRG 50 % + LPS. b: L-PRG 50 % + LPS and P-PRG 50 % + LPS. c: P-PRG 25 % + LPS. **b** Significantly different with a: P-PRG 50 % + LPS. B) b: L-PRG 50 % + LPS and P-PRG 50 % + LPS. f: L-PRG 50 % + LPS. c: P-PRG 50 % + LPS. d: P-PRG 25 % + LPS. *Denote significant differences (p < 0.01) between the same variable at 1 h and 48 h and at 49 h and 96 by t-paired test. Data are presented as means (mean standard errors [m.s.e])

Synovial fluid, L-PRG and P-PRG supernatants, and culture media alone or with PRG supernatants obtained at 1, 48, 49 and 96h were used to determine the concentration of PDGF-BB, TGF-β_1_, TNF-α, IL-4, IL-1ra and HA via ELISA by duplicate (Fig. 2). All proteins and HA were assayed using commercial ELISA development kits from R&D Systems (Minneapolis, MN, USA). PDGF-BB (Human PDGF-BB DuoSet, DY220) and TGF-β_1_ (Human TGF-β1 DuoSet, DY240E) were determined using human antibodies because there is a high homology between these proteins in humans and horses [31, 32]. Furthermore, these kits have been used for the same purposes in other equine PRP studies [28]. TNF-α (Equine TNF-alpha DuoSet, DY1814), IL-4 (Equine IL-4 DuoSet, DY1809) and IL-1ra (Equine IL-1ra/IL-1F3 DuoSet, DY1814) were assayed with equine-specific antibodies, and HA (Hyaluronan, DuoSet, DY3614) was determined using a multispecies detection ELISA kit. The standards provided for each ELISA kit were used in preparing each standard curve according to the manufacturers’ instructions. Readings were performed at 450 nm.

### Statistical and data analysis

The statistical analysis was performed with the software SPSS 19.0 (IBM, Chicago, IL, USA). The Shapiro–Wilk test was used to assess the fit of the data set to a normal distribution (goodness of fit). Both PLT and WBC counts in whole blood and both PRP presented a normal distribution (p > 0.05). PDGF-BB, TGF-β_1_, TNF-α, IL-4, IL-1ra and HA concentrations were normalized using logarithm (Y) transformations.

Platelet and WBC counts in whole blood and both PRP preparations were evaluated through a one-way analysis of variance (ANOVA), followed by a Tukey test. PDGF-BB, TGF-β_1_, TNF-α, IL-4, IL-1ra and HA concentrations from both PRG supernatants and synovial fluid were evaluated in a similar fashion to blood cells. PDGF-BB, TGF-β_1_, TNF-α, IL-4, IL-1ra and HA concentrations in culture media obtained at 48 and 96h from LPS-challenged non-challenged SME groups cultured with 25 and 50 % P-PRG and L-PRG supernatants, respectively, were evaluated using a generalized lineal model (GLM), followed by a Tukey test when necessary.

The initial PDGF-BB, TGF-β_1_, TNF-α, IL-4, IL-1ra and HA concentrations in fresh culture media with PRG supernatants at 1 and 48h were also considered in the analysis. However, data regarding the PDGF-BB, TGF-β_1_, TNF-α, IL-4, IL-1ra and HA concentrations obtained at these two periods were compared with the concentrations of these proteins and HA at 48 and 96h, respectively, using a t-paired test. A correlation analysis was performed in order to determine the Pearson correlation product (*r*) between the variables evaluated in the study. A p-value < 0.05 was accepted as statistically significant for all tests. Data are presented as mean ± mean standard error (m.s.e).

## Results

### Cell concentration in L-PRP and P-PRP

Platelet counts were significantly different between whole blood, L-PRP and P-PRP, with P-PRP having the lowest concentration (×0.8), followed by whole blood (×1) and L-PRP (×2.5). WBC counts were also significantly different between the groups evaluated, with L-PRP having the highest concentration (×4), followed by whole blood (×1) and P-PRP (×0.15) (Table 1).

**Table 1:** Concentrations of the cells and molecules evaluated in whole blood, both platelet rich gel (PRG) supernatants and synovial fluid^a^

	Fluid			
Variable	Whole blood	L-PRG	P-PRG	Synovial fluid
Platelet ×10^3^/μL	124.7 ± 3.1	311.6 ± 20.4^b^	99.4 ± 4.3^c^	ND
WBC ×10^3^/μL	8.4 ± 3.6	34.2 ± 3.7^b^	0.13 ± 0.03^c^	ND
RBC ×10^6^//μL	6.31 ± 0.3	1.6 ± 0.4^b^	0.02 ± 0.01^c^	ND
TGF-β_1_ (pg/mL)	ND	1669.2 ± 313.2	1369.2 ± 21.4	1413.8 ± 4.8
PDGF-BB (pg/mL)	ND	3069. 9 ± 1261.6	383.8 ± 80.9^b^	60.5 ± 0.9^b^
TNF-α (pg/mL)	ND	60 ± 0.5^b,c^	59 ± 1.4^b^	66.7 ± 3.3^c^
IL-4 (pg/mL)	ND	75.7 ± 9.3^b^	61.1 ± 1.52^b^	101.8 ± 33.7^c^
IL-1ra (pg/mL)	ND	160.4 ± 68.0	58.7 ± 3.1^b^	77.8 ± 10.7
Hyaluronic acid (ng/mL)	ND	6.9 ± 2.9	2.3 ± 1.08	53017.6 ± 12140^b^

^a^ Data are presented as means (m.s.e).^b-c^ Lowercase letters denote significant differences (p < 0.01) between groups in the same row by Tukey test. ND: no determined

### Concentration of growth factors, cytokines and HA in L-PRP/L-PRG, P-PRP/P-PRG, synovial fluid and culture media of SME

#### TGF-β_1_

TGF-β_1_ concentration was similar between L-PRG, P-PRG and synovial fluid (Table 1). The concentration of TGF-β_1_ increased over time in each of the groups evaluated, even in the control groups (either with or without LPS). TGF-β_1_ concentrations were significantly higher (p < 0.01) in culture media produced from SME cultured with 50 % P-PRG supernatant than they were in media produced from SME cultured with 25 % P-PRG supernatant. However, no other significant differences were observed at the commencement of the experiment (1h) or at the second culture media change (49h) (Fig. 2a-b).

At 48h, the TGF-β_1_ concentration in all SME groups increased significantly (p < 0.01) in comparison to that recorded at the start of the experiment (1h). The TGF-β_1_ concentration was significantly (p < 0.01) higher in the SME group with 50 % L-PRG supernatant as compared to the SME control group without LPS, SME cultured with 25 % L-PRG and 25 % P-PRG supernatants. The SME cultured with 25 % P-PRG supernatant presented with a significantly lower (p < 0.01) concentration of TGF-β_1_ as compared to all PRG supernatant treated groups and synovial fluid. The TGF-β_1_ concentration was similar across SME control groups (with and without LPS) (Fig. 2a).

At 96h, a significant increase (p < 0.01) in the concentration of TGF-β_1_ was observed in the SME group cultured with 50 % L-PRG supernatant as compared with SME control groups with and without LPS, and the SME group cultured with 25 % P-PRG supernatant. It is noteworthy that at 48 and 96h, the concentration of TGF-β_1_ in the evaluated SME groups was similar to that of synovial fluid, the SME control group without LPS being the exception (Fig. 2b).

#### PDGF-BB

**Fig. 3:**
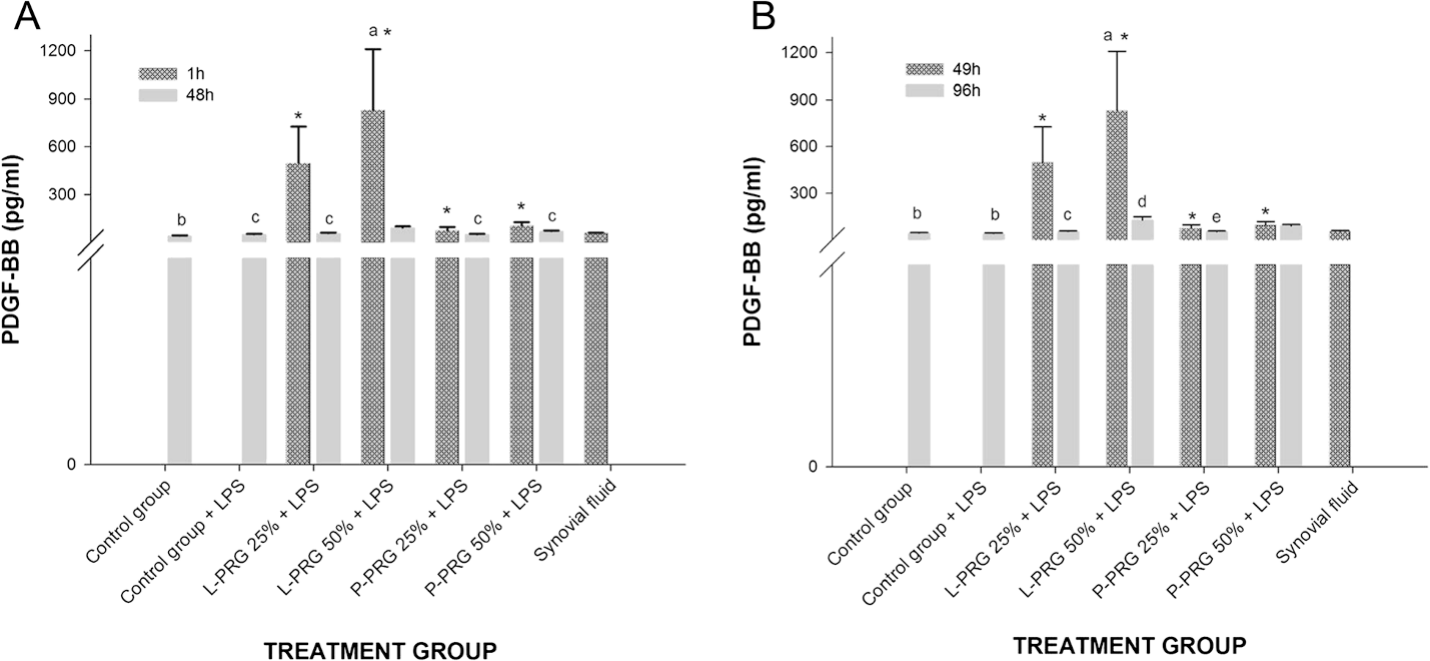
PDGF-BB concentrations obtained in culture media of the synovial membrane explant groups at 48 (**a**) and 96 h (**b**). ^a-b^ Lowercase letters denote significant (p < 0.01) differences between groups in the same column by Tukey test. **a** Significantly different with a: P-PRG 25 % + LPS. b: L-PRG 50 % + LPS and P-PRG 50 % + LPS. c: L-PRG 50 % + LPS. **b** Significantly different with a: P-PRG 25 % + LPS. b: L-PRG 50 % + LPS and P-PRG 50 % + LPS. c: SD from L-PRG 50 % + LPS. d: P-PRG 25 % + LPS. e: P-PRG 50 % + LPS. *Denote significant differences (p < 0.01) between the same variable at 1 h and 48 h and at 49 h and 96 by t-paired test. Data are presented as means (m.s.e)

L-PRG presented a significantly (p < 0.01) higher PDGF-BB concentration than did P-PRG and synovial fluid (Table 1). PDGF-BB concentrations at 1 and 49h were significantly higher (p < 0.01) in the SME groups cultured with 50 % L-PRG supernatant than they were in the other SME groups. At 48h, an important diminution of PDGF-BB concentration was observed in all SME groups treated with the PRG supernatants (Fig. 3a-b).

At 48h, the SME group cultured with 50 % L-PRG supernatant presented with a significantly higher (p < 0.01) concentration of PDFG-BB than did all other groups evaluated (Fig. 3a). At 96h a similar trend in the concentration of PDGF-BB was observed. Note that PDFG-BB was produced by the SME without the addition of any PRG supernatant; moreover, at all times, the concentration of PDGF-BB remained similar among all of the treated groups and synovial fluid (Fig. 3b).

#### TNF-α

**Fig. 4:**
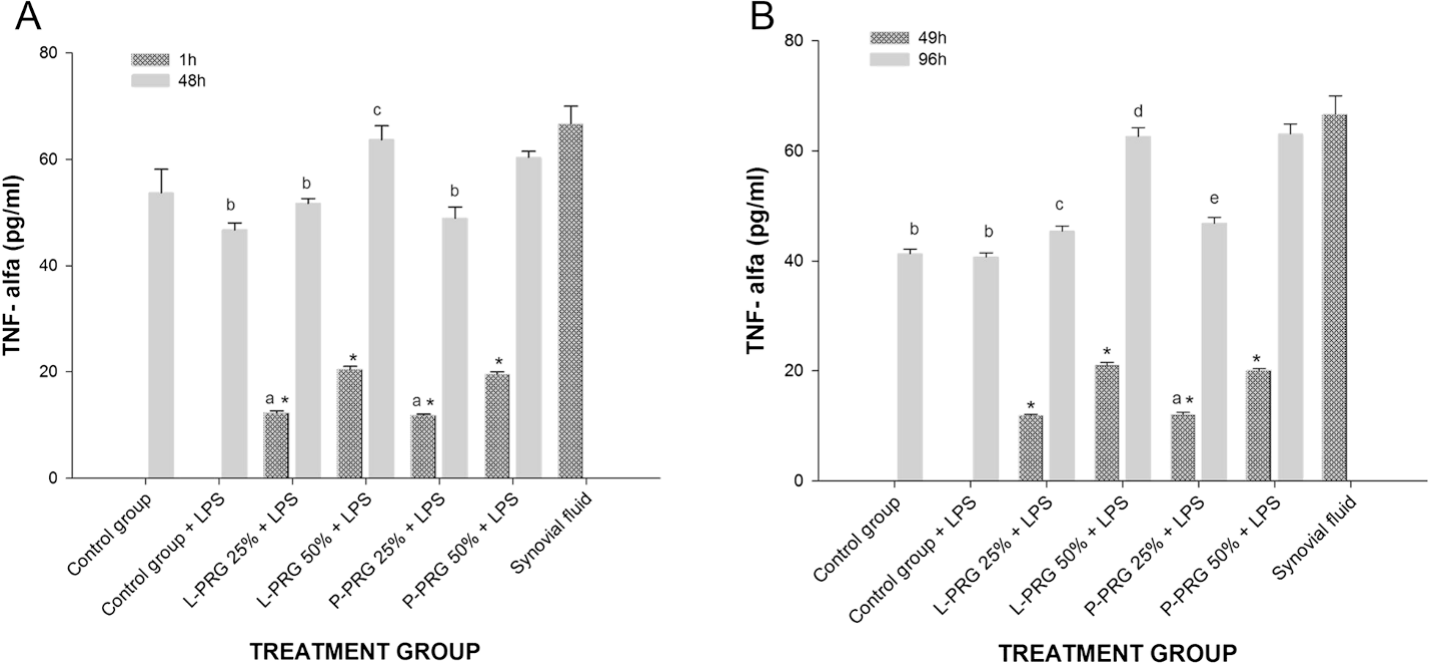
TNF-α concentrations obtained in culture media of the synovial membrane explant groups at 48 (**a**) and 96 h (**b**). ^a-b^ Lowercase letters denote significant (p < 0.01) differences between groups in the same column by Tukey test. **a** Significantly different with a: L-PRG 50 % + LPS and P-PRG 50 % + LPS. b: L-PRG 50 % + LPS and P-PRG 50 % + LPS. c: P-PRG 25 % + LPS. **b** Significantly different with a: L-PRG 50 % + LPS and P-PRG 50 % + LPS. b: L-PRG 50 % + LPS, P-PRG 25 % + LPS and P-PRG 50 % + LPS. c: L-PRG 50 % + LPS and P-PRG 50 % + LPS. d: P-PRG 25 % + LPS. e: P-PRG 50 % + LPS. *Denote significant differences (p < 0.01) between the same variable at 1 h and 48 h and at 49 h and 96 by t-paired test. Data are presented as means (m.s.e)

Synovial fluid demonstrated the highest (p < 0.01) TNF-α concentration, whereas low concentrations for this cytokine were observed in both P-PRG supernatants (Table 1). The TNF-α concentration was significantly higher (p < 0.01) at 1 and 49h in the SME groups cultured with both 50 % PRG supernatants (Fig. 4a-b). However, the TNF-α concentration in all SME groups, including those that did not receive any PRG supernatant, was significantly higher at 48 and 96h than at 1 or 49 h. At 48h, the TNF-α concentration was significantly higher (p < 0.01) in the SME groups cultured with 50 % PRG supernatants than in either the control groups or SME groups cultured with both 25 % PRG supernatants (Fig. 4a). At 96h, the TNF-α concentration was significantly lower in both SME control groups than it was in both concentrations of L-PRG supernatants and the 50 % P-PRG supernatant. TNF-α concentrations were similar between SME groups cultured with both 50 % PRG supernatants and synovial fluid at 48 and 96h (Fig. 4b).

#### IL-4

**Fig. 5:**
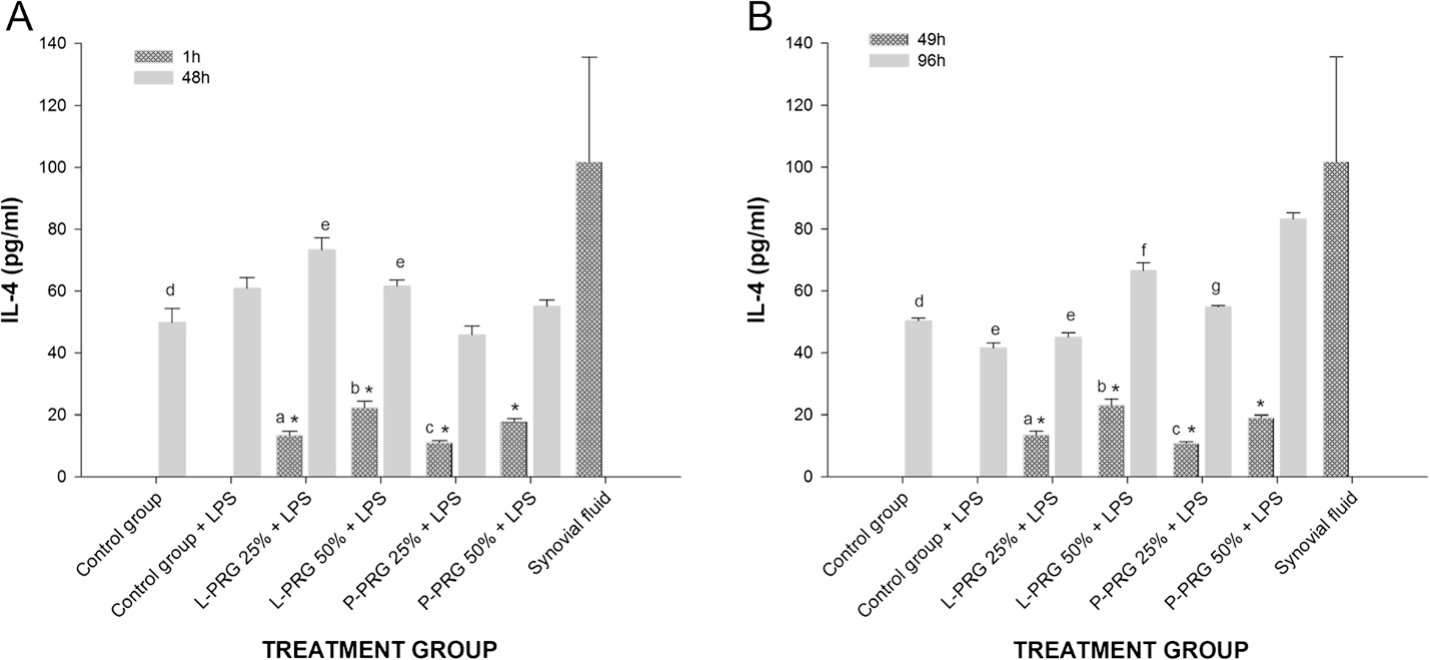
IL-4 concentrations obtained in culture media of the synovial membrane explant groups at 48 (**a**) and 96 h (**b**). ^a-b^ Lowercase letters denote significant (p < 0.01) differences between groups in the same column by Tukey test. **a** Significantly different with a: a: L-PRG 50 % + LPS. b: P-PRG 25 % + LPS. c: P-PRG 50 % + LPS. d: L-PRG 25 % + LPS. e: P-PRG 25 % + LPS. **b** Significantly different with a: L-PRG 50 % + LPS. b: P-PRG 25 % + LPS. c: P-PRG 50 % + LPS. d: Control group + LPS, L-PRG 50 % + LPS and P-PRG 50 % + LPS. e: L-PRG 50 % + LPS, P-PRG 25 % + LPS and P-PRG 50 % + LPS. f: P-PRG 25 % + LPS and P-PRG 50 % + LPS. g: SD from P-PRG 50 % + LPS. *Denote significant differences (p < 0.01) between the same variable at 1 h and 48 h and at 49 h and 96 by t-paired test. Data are presented as means (m.s.e)

Synovial fluid demonstrated the highest (p < 0.01) IL-4 concentration, whereas low concentrations for this cytokine were observed in both P-PRG supernatants (Table 1). At 1 and 49h, the IL-4 concentration was significantly higher (p < 0.01) in the culture media of the SME groups cultured with the 50 % PRG supernatants than it was in those cultured with the 25 % PRG supernatants (Fig. 5a-b). At 48h, the IL-4 concentration was significantly higher (p < 0.01) in the culture media of the SME groups treated with either 25 % or 50 % L-PRG supernatants as compared to the SME group treated with the 25 % P-PRG supernatant. At this time, the SME control group without LPS exhibited a significantly lower (p < 0.01) concentration of IL-4 as compared to the SME group treated with the 25 % L-PRG supernatant (Fig. 5a). At 96h a marked drop in the IL-4 concentration was observed in the SME control group with LPS as compared to the SME control group without LPS and the SME groups treated with both 50 % L-PRG and P-PRG supernatants. At 48 and 96h, the concentration of IL-4 was significantly higher (p < 0.01) in synovial fluid than in the evaluated groups, with the exception of the SME treated with the 50 % P-PRG supernatant. The IL-4 concentration in the SME treated with 50 % P-PRG supernatant was similar to that found in the synovial fluid (Fig. 5b).

#### IL-1ra

**Fig. 6:**
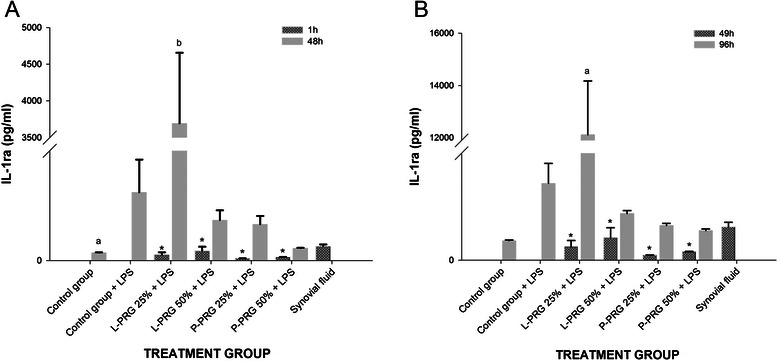
IL-1ra concentrations obtained in culture media of the synovial membrane explant groups at 48 (**a**) and 96 h (**b**). ^a-b^ Lowercase letters denote significant (p < 0.01) differences between groups in the same column by Tukey test. **a** Significantly different with a: all groups. b: all groups. **b** Significantly different with a: all groups. *Denote significant differences (p < 0.01) between the same variable at 1 h and 48 h and at 49 h and 96 by t-paired test. Data are presented as means (m.s.e)

L-PRG supernatants and synovial fluid presented significantly higher (p < 0.01) IL-1ra concentrations than P-PRG supernatants did (Table 1). Temporally, the IL-1ra concentration remained consistently and significantly higher (p < 0.01) in the SME group treated with 25 % L-PRG supernatant as compared to all other experimental groups, synovial fluid and P-PRG supernatants. IL-1ra increased 10 to 30 times more in the SME group than in any other group. Interestingly, treating the SME group with 50 % L-PRG inhibited the production of IL-1ra (Fig. 6a-b).

#### HA

**Fig. 7:**
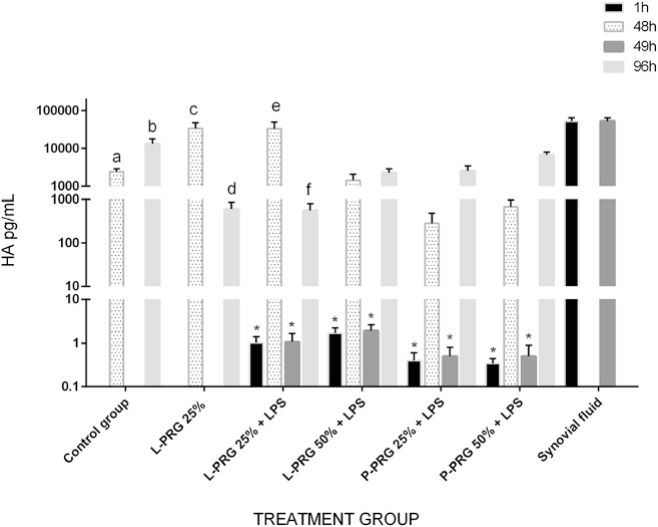
HA concentrations obtained in culture media of the synovial membrane explant groups at 48 and 96 h. ^a-b^ Lowercase letters denote significant (p < 0.01) differences between groups in the same column by Tukey test. Significantly different with a: Control group + LPS and L-PRG 25 % + LPS. b: L-PRG 50 % + LPS, P-PRG 25 % + LPS and P-PRG 50 % + LPS. c: L-PRG 50 % + LPS, P-PRG 25 % + LPS and P-PRG 50 % + LPS. d: Control group + LPS and L-PRG 25 % + LPS. e: P-PRG 25 % + LPS and P-PRG 50 % + LPS. f: P-PRG 50 % + LPS. *Denote significant differences (p < 0.01) between the same variable at 1h and 48 h and at 49 h and 96 by t-paired test. Data are presented as means (m.s.e)

HA concentration was significantly (p < 0.01) higher in synovial fluid than both PRG supernatants (Table 1). At 1 and 49h, the HA concentration was significantly lower (p < 0.01) in the culture media of all SME groups treated with PRG supernatants. At 48h, there was a significant increase (p < 0.01) in the concentration of HA in the SME control group with LPS and the SME group treated with 25 % L-PRG supernatant as compared to the SME control group without LPS and the SME groups treated with other PRG supernatants. At 96h, a significant decrease (p < 0.01) in HA concentration was observed in both the SME control group with LPS and those treated with 25 % L-PRG supernatant as compared to the SME control group without LPS. However, the HA concentration was similar for the SME control group without LPS and the SME groups treated with 50 % L-PRG supernatant and with 25 and 50 % P-PRG supernatants; however, in this last group, the HA concentration was apparently higher than in the SME group cultured with 50 % L-PRG supernatant. In contrast, the concentration of HA was significantly higher (p < 0.01) in synovial fluid than it was in all other SME groups at any time (Fig. 7).

### Correlations

At 48h, TGF-β_1_ and PDGF-BB (*r* = 0.69, p = 0.0001), and IL-1ra and HA (*r* = 0.76, p = 0.0001), were found to be significantly correlated. Moreover, at 96h, TGF-β_1_ and PDGF-BB (*r* = 0.69, p = 0.0001), PDGF-BB and TNF-α (*r* = 0.71, p = 0.0001), PDGF-BB and IL-4 (*r* = 0.67, p = 0.0001), and TNF-α and IL-4 (*r* = 0.81, p = 0.0001) were found to be significantly correlated.

## Discussion

This study investigated the *in vitro* effects of two concentrations (25 and 50 %) of L-PRG and P-PRG supernatants on SME conditioned with LPS. The results indicate that both L-PRP and P-PRP supernatants exert an anti-inflammatory (or regulatory) and anabolic mechanism in an *in vitro* LPS-induced synovial membrane inflammation.

The molecules evaluated in this study were selected because they have been implicated in the pathophysiology of OA [19]. TGF-β_1_ and PDGF-BB were assayed because they have demonstrated an important anabolic/anti-inflammatory action on synovial membrane and cartilage [33]. Both GF increase the production of cartilage extracellular matrix (ECM) proteins, decrease both joint pain and inflammation and promote the differentiation of synovial membrane cells in chondrocytes [19, 33]. Both proteins are stored mainly in platelet alpha granules. For, this reason, many of the therapeutic effects of PRP have been attributed to these proteins [5].

TNF-α was selected as a pro-inflammatory cytokine because this protein and IL-1 represent some of the key cytokines associated with the catabolic state found in OA [34]. TNF-α is implicated in synovitis, and its up-regulation in synovial tissue is associated with a more aggressive clinical picture of erosive arthritis [35]. Furthermore, a clinical study revealed that this cytokine is a more useful biomarker for discriminating OA severity in horses than is IL-1 [36].

IL-1ra and IL-4 were selected because they have an important anti-inflammatory effect and because they are up-regulated in OA patients [37, 38]. IL-1ra is a natural antagonist of IL-1 effects because it blocks the cellular receptors necessary for inducing joint inflammation and cartilage catabolism mediated by this last cytokine [39]. IL-4 is associated with chondroprotection because it increases the synthesis of cartilage ECM [40]. This protein is more anti-inflammatory than anabolic because it increases the synthesis of IL-1ra and down-regulates TNF-α [41].

We decided to measure the concentration of these molecules in synovial fluid to determine which of the two concentrations of the PRG supernatants could reflect a near or similar apparently healthy molecular synovial fluid environment in the culture media of the SME groups challenged with LPS. Notably, both PRG supernatants induced concentrations of GF, cytokine and HA very similar to those found in synovial fluid when compared with the SME control groups (either with LPS or without LPS). However, every PRG supernatant and its respective concentration produced different synovial membrane responses in the molecules evaluated in the study, which could indicate that different mechanisms of action underlie the anti-inflammatory and anabolic effects depending on the cellular and molecular profile of the PRP/PRG supernatant used in the study.

The two most attractive PRG supernatants and concentrations for exploring the *in vivo* effects of these substances, whether in an animal model of OA or in the clinical setting, were 25 % L-PRG and 50 % P-PRG supernatants. At a concentration of 25 %, the L-PRG supernatant produced the highest IL-1ra concentration in the culture media from SME when compared with the rest of the SME groups treated with both PRG at different concentrations. IL-1ra has been evaluated experimentally and clinically as a treatment for OA on the basis of its capacity to inhibit the binding of both IL-1α and IL-1β to their active receptor [42]. However, there are contradictory results from clinical trials, in which this purified protein had little success, probably because there was sufficient IL-1ra already in the OA synovial fluid or simply because IL-1ra did not work well in OA in the context of an inflammatory milieu [43].

Furthermore, the 25 % L-PRG supernatant produced an extensive depression in HA synthesis in the SME at 96h. This intriguing situation could be explained by way of an inhibitory mechanism in HA production as mediated by a higher concentration of IL-1ra. However, to our knowledge, this mechanism has not been previously reported, although it is known that IL-1ra does not affect HA synthesis in fibroblasts *in vitro* [44]. Moreover, it is important to consider that patients with cranial cruciate knee injury who were treated with IL-1ra (anakinra) demonstrated better clinical outcomes and a significant decrease in plasma HA than did the placebo group [45]. However, *in vitro* research has demonstrated that IL-1ra prevents cartilage ECM explant degradation [46]. Our results, in conjunction with the aforementioned study, could indicate that IL-1ra, or associated therapies such as IRAP or autologous conditioned serum, can provide relief from joint inflammation after injury, but does not induce joint anabolism [47].

The elevated and sustained concentration of IL-4 over time in the 50 % P-PRG supernatant indicated an anti-inflammatory profile. IL-4 is an anti-inflammatory cytokine associated with the downregulation of IL-1β and matrix metalloproteinases 2, 9 and 13 in SME [48]. In addition, HA production was sustained in response to the 50 % P-PRG supernatant, but depressed in both the SME control group with LPS and the SME group cultured with 25 % L-PRG supernatant. This desirable anabolic effect, observed in the 50 % P-PRG supernatant, has been observed previously *in vitro*, in which synoviocytes from OA patients were cultured with P-PRP [15]. Both platelet TGF-β_1_ and PDGF-BB have been shown to induce the synthesis of HA through the up-regulation of HA synthase isoform 2 [49].

Although a statistical correlation does not always imply a direct biological interaction, we observed important correlations that could potentially be useful for demonstrating the complexity of the biological interactions observed in this *in vitro* system of synovial inflammation and the biological responses of the SME challenged with LPS to the PRG supernatants. The correlation observed between TGF-β_1_ and PDGF-BB is explained by way of GF being stored and released from PLT in PRP [28]. The strong correlation (0.76) between IL-1ra and HA at 48h suggests that a molecular mechanism, mediated by IL-1ra, induced strong HA production in the first 48h, but that this cytokine might also be implicated in a later HA production inhibitory mechanism. This metabolic change was most pronounced in the culture media of the SME groups cultured with 25 % L-PRG supernatant at 96h.

Other important and possibly chained biological correlations were observed at 96h between PDGF-BB and TNF-α, PDGF-BB and IL-4, and TNF-α and IL-4. These correlations could indicate the importance of PDGF-BB in the production or up-regulation of the anti-inflammatory cytokine IL-4. On the other hand, the results from this study demonstrate that at least in this *in vitro* system of synovial membrane inflammation, TNF-α may act more like a regulatory than a pro-inflammatory cytokine [50]. This finding could be controversial, given the results observed in animal models and in patients with natural synovial inflammatory diseases. In such cases, the increased concentration or up-regulation of TNF-α correlates with the severity of the synovial membrane inflammation and, consequently with articular cartilage degradation [36, 46, 51].

The results of this study support the hypothesis that some PRP preparations could be indicated for the treatment of patients with OA. It is also important to note that the higher concentration of PLTs in PRP is not crucial, as previously indicated by Weibrich et al. [52]. Our results support the findings of several previous tendon explant studies by demonstrating that in order to induce anabolic signalling, it is more important to reduce the WBC concentrations than it is to increase the PLT concentration in PRP [24, 25].

The present study had several limitations, mainly associated with the fact that animal models of joint disease are better than *in vitro* studies in capturing the biological phenomena in patients with naturally occurring OA or inflammatory synovitis. As mentioned by Andia and Maffulli [53], the current *in vitro* systems of joint disease are not able to address the role of the immunologic system, notably the macrophage activity induced by proteins of the group-specific component globulin that activates these cells via the innate immune toll-like receptor 4 and induces the expression of TNF-α, IL-1β, IL-6, and vascular endothelial growth factor. Therefore, *in vitro* studies are not fully able to reflect the real *in vivo* joint disease and its response to treatments [53]. On the other hand, we used the blood of a single animal for culturing the SME of different horses; this situation is not ideal in clinical conditions, but it is advantageous in *in vitro* studies because the cellular product evaluated can be standardized for cell and protein concentrations, which reduces the variability in the biological response of the tissues treated experimentally. Finally, many GF and cytokines implicated in the genesis of OA, mainly IL-1 and IL-6 [37], were not measured in this study; thus, it is not possible to describe the actual effect of PRP on these proteins and their interaction with the molecules evaluated.

## Conclusions

The results of this study lead to the conclusion that both L-PRG and P-PRG supernatants at different concentrations produce various inflammatory, anti-inflammatory and anabolic responses in SME conditioned with LPS, which confirmed the working hypothesis. Although the 25 % L-PRG supernatant induced the highest concentration of IL-1ra in the culture media from the SME conditioned with LPS at 48 and 96h, it produced a strong metabolic depression in the production of HA at 96h. In contrast, the 50 % P-PRG supernatant produced a more sustained concentration of IL-4 and a significant increase in the production of HA during that period of time. These *in vitro* findings suggest that the anabolic and anti-inflammatory joint responses will depend on the leukocyte and platelet concentration of the PRP preparation and on the volume of this substance injected. Moreover, it is possible that leukoreduced PRP preparations are more effective in the medical treatment of patients with OA and inflammatory synovitis. However, additional *in vivo* studies, evaluating the effect of both L-PRP and P-PRP in either animal models or in patients are necessary.

## Abbreviations

ANOVA: Analysis of variance
DMEM: Dulbecco’s Modified Eagle Medium
ELISA: Enzyme-linked immunosorbent assay
GF: Growth factor
HA: Hyaluronan
IL (IL-1b: IL-4 and IL-6), Interleukin
IL-1ra: IL-1 antagonist receptor
WBC: Leukocyte
L-PRG: Leukoconcentrated platelet-rich gel
L-PRP: Leukoconcentrated platelet-rich plasma
LPS: Lipopolysaccharide
OA: Osteoarthritis
PLT: Platelet
PDGF-BB: Platelet derived-growth factor isoform BB
PRG: Platelet-rich gel
PRP: Platelet-rich plasma
P-PRG: Pure platelet-rich gel
P-PRP: Pure platelet-rich plasma
RBC: Red blood cell
SME: Synovial membrane explants
TGF-β_1_: Transforming growth factor beta-1
TNF-α: Tumor necrosis factor alpha

## References

[1] Abhishek A, Doherty M (2013). Diagnosis and clinical presentation of osteoarthritis. Rheum Dis Clin North Am.

[2] Sokolove J, Lepus CM (2013). Role of inflammation in the pathogenesis of osteoarthritis: latest findings and interpretations. Ther Adv Musculoskelet Dis.

[3] Sofat N, Beith I, Anilkumar PG, Mitchell P (2011). Recent clinical evidence for the treatment of osteoarthritis: what we have learned. Rev Recent Clin Trials.

[4] Filardo G, Kon E, Di Martino A, Di Matteo B, Merli ML, Cenacchi A, Fornasari PM, Marcacci M (2012). Platelet-rich plasma vs hyaluronic acid to treat knee degenerative pathology: study design and preliminary results of a randomized controlled trial. BMC Musculoskelet Disord.

[5] Filardo G, Kon E, Roffi A, Di Matteo B, Merli ML, Marcacci M. Platelet-rich plasma: why intra-articular? A systematic review of preclinical studies and clinical evidence on PRP for joint degeneration. Knee Surg Sports Traumatol Arthrosc 2013: doi:10.1007/s00167-00013-02743-00161. 10.1007/s00167-013-2743-1 PMC4541701 24275957

[6] Andia I, Abate M (2014). Knee osteoarthritis: Hyaluronic acid, platelet-rich plasma or both in association?. Expert Opin Biol Ther.

[7] Wang-Saegusa A, Cugat R, Ares O, Seijas R, Cuscó X, Garcia-Balletbó M (2011). Infiltration of plasma rich in growth factors for osteoarthritis of the knee short-term effects on function and quality of life. Arch Orthop Trauma Surg.

[8] Carmona JU, Argüelles D, Climent F, Prades M (2007). Autologous platelet concentrates as a treatment of horses with osteoarthritis: a preliminary pilot clinical study. J Equine Vet Sci.

[9] Pichereau F, Décory M, Cuevas Ramos G (2014). Autologous platelet concentrate as a treatment for horses with refractory fetlock osteoarthritis. J Equine Vet Sci.

[10] Silva RF, Carmona JU, Rezende CMF (2013). Intra-articular injections of autologous platelet concentrates in dogs with surgical reparation of cranial cruciate ligament rupture. Vet Comp Orthop Traumatol.

[11] Kisiday JD, McIlwraith CW, Rodkey WG, Frisbie DD, Steadman JR (2012). Effects of platelet-rich plasma composition on anabolic and catabolic activities in equine cartilage and meniscal explants. Cartilage.

[12] Van Buul GM, Koevoet WLM, Kops N, Bos PK, Verhaar JAN, Weinans H, Bernsen MR, Van Osch GJVM (2011). Platelet-rich plasma releasate inhibits inflammatory processes in osteoarthritic chondrocytes. Am J Sports Med.

[13] Amrichová J, Špaková T, Rosocha J, Harvanová D, Bačenková D, Lacko M, Horňák S (2014). Effect of PRP and PPP on proliferation and migration of human chondrocytes and synoviocytes in vitro. Central Eur J Biol.

[14] Sundman EA, Cole BJ, Karas V, Della Valle C, Tetreault MW, Mohammed HO, Fortier LA (2014). The anti-inflammatory and matrix restorative mechanisms of platelet-rich plasma in osteoarthritis. Am J Sports Med.

[15] Anitua E, Sánchez M, Nurden AT, Zalduendo MM, De la Fuente M, Azofra J, Andía I (2007). Platelet-released growth factors enhance the secretion of hyaluronic acid and induce hepatocyte growth factor production by synovial fibroblasts from arthritic patients. Rheumatology.

[16] Lippross S, Moeller B, Haas H, Tohidnezhad M, Steubesand N, Wruck CJ, Kurz B, Seekamp A, Pufe T, Varoga D (2011). Intraarticular injection of platelet-rich plasma reduces inflammation in a pig model of rheumatoid arthritis of the knee joint. Arthritis Rheum.

[17] Mastrangelo AN, Vavken P, Fleming BC, Harrison SL, Murray MM (2011). Reduced platelet concentration does not harm PRP effectiveness for ACL repair in a porcine in vivo model. J Orthop Res.

[18] Textor JA, Willits NH, Tablin F (2013). Synovial fluid growth factor and cytokine concentrations after intra-articular injection of a platelet-rich product in horses. Vet J.

[19] Fortier LA, Barker JU, Strauss EJ, McCarrel TM, Cole BJ (2011). The role of growth factors in cartilage repair. Clin Orthop Relat Res.

[20] Chang KV, Hung CY, Aliwarga F, Wang TG, Han DS, Chen WS (2014). Comparative effectiveness of platelet-rich plasma injections for treating knee joint cartilage degenerative pathology: a systematic review and meta-analysis. Arch Phys Med Rehabil.

[21] Cavallo C, Filardo G, Mariani E, Kon E, Marcacci M, Pereira Ruiz MT, Facchini A, Grigolo B (2014). Comparison of platelet-rich plasma formulations for cartilage healing: an in vitro study. J Bone Joint Surg Am.

[22] Carmona JU, López C, Sandoval JA (2013). Review of the currently available systems to obtain platelet related products to treat equine musculoskeletal injuries. Rec Pat Reg Med.

[23] Dohan Ehrenfest DM, Bielecki T, Mishra A, Borzini P, Inchingolo F, Sammartino G, Rasmusson L, Everts PA (2012). In search of a consensus terminology in the field of platelet concentrates for surgical use: Platelet-Rich Plasma (PRP), Platelet-Rich Fibrin (PRF), fibrin gel polymerization and leukocytes. Curr Pharm Biotechnol.

[24] McCarrel TM, Minas T, Fortier LA (2012). Optimization of leukocyte concentration in platelet-rich plasma for the treatment of tendinopathy. J Bone Joint Surg Am.

[25] Boswell SG, Schnabel LV, Mohammed HO, Sundman EA, Minas T, Fortier LA (2014). Increasing platelet concentrations in leukocyte-reduced platelet-rich plasma decrease collagen gene synthesis in tendons. Am J Sports Med.

[26] Braun HJ, Kim HJ, Chu CR, Dragoo JL (2014). The effect of platelet-rich plasma formulations and blood products on human synoviocytes: Implications for intra-articular injury and therapy. Am J Sports Med.

[27] Dhillon RS, Schwarz EM, Maloney MD (2012). Platelet-rich plasma therapy - future or trend?. Arthritis Res Ther.

[28] Giraldo CE, López C, Álvarez ME, Samudio IJ, Prades M, Carmona JU (2013). Effects of the breed, sex and age on cellular content and growth factor release from equine pure-platelet rich plasma and pure-platelet rich gel. BMC Vet Res.

[29] Argüelles D, Carmona JU, Pastor J, Iborra A, Viñals L, Martínez P, Bach E, Prades M (2006). Evaluation of single and double centrifugation tube methods for concentrating equine platelets. Res Vet Sci.

[30] Moses VS, Hardy J, Bertone AL, Weisbrode SE (2001). Effects of anti-inflammatory drugs on lipopolysaccharide-challenged and -unchallenged equine synovial explants. Am J Vet Res.

[31] Donnelly BP, Nixon AJ, Haupt JL (2006). Nucleotide structure of equine platelet-derived growth factor-A and –B and expression in horses with induced acute tendinitis. Am J Vet Res.

[32] Penha-Goncalves MN, Onions DE, Nicolson L (1997). Cloning and sequencing of equine transforming growth factor-beta 1 (TGFβ-1) cDNA. DNA Seq.

[33] Civinini R, Nistri L, Martini C, Redl B, Ristori G, Innocenti M (2013). Growth factors in the treatment of early osteoarthritis. Clin Cases Min Bone Metab.

[34] Miller RE, Miller RJ, Malfait A-M (2014). Osteoarthritis joint pain: the cytokine connection. Cytokine.

[35] Eder L, Thavaneswaran A, Chandran V, Gladman DD (2014). Tumour necrosis factor α blockers are more effective than methotrexate in the inhibition of radiographic joint damage progression among patients with psoriatic arthritis. Ann Rheum Dis.

[36] Kamm JL, Nixon AJ, Witte TH (2010). Cytokine and catabolic enzyme expression in synovium, synovial fluid and articular cartilage of naturally osteoarthritic equine carpi. Equine Vet J.

[37] Carmona JU, Prades M (2009). Pathophisiology of osteoarthritis. Comp Equine.

[38] Fernandes JC, Martel-Pelletier J, Pelletier J-P (2002). The role of cytokines in osteoarthritis pathophysiology. Biorheology.

[39] Yang KGA, Raijmakers NJH, van Arkel ERA, Caron JJ, Rijk PC, Willems WJ, Zijl JAC, Verbout AJ, Dhert WJA, Saris DBF (2008). Autologous interleukin-1 receptor antagonist improves function and symptoms in osteoarthritis when compared to placebo in a prospective randomized controlled trial. Osteoarthritis Cartilage.

[40] Millward-Sadler SJ, Wright MO, Davies LW, Nuki G, Salter DM (2000). Mechanotransduction via integrins and interleukin-4 results in altered aggrecan and matrix metalloproteinase 3 gene expression in normal, but not osteoarthritic, human articular chondrocytes. Arthritis Rheum.

[41] Lee S-G, Lee E-J, Park W-D, Kim J-B, Kim E-O, Choi S-W (2012). Anti-inflammatory and anti-osteoarthritis effects of fermented Achyranthes japonica Nakai. J Ethnopharmacol.

[42] Furman BD, Mangiapani DS, Zeitler E, Bailey KN, Horne PH, Huebner JL, Kraus VB, Guilak F, Olson SA (2014). Targeting pro-inflammatory cytokines following joint injury: acute intra-articular inhibition of interleukin-1 following knee injury prevents post-traumatic arthritis. Arthritis Res Ther.

[43] Malemud C (2010). Anticytokine therapy for osteoarthritis. Drugs Aging.

[44] Cao HJ, Wang HS, Zhang Y, Lin HY, Phipps RP, Smith TJ (1998). Activation of human orbital fibroblasts through CD40 engagement results in a dramatic induction of hyaluronan synthesis and prostaglandin endoperoxide H synthase-2 expression: Insights into potential pathogenic mechanisms of thyroid-associated ophthalmopathy. J Biol Chem.

[45] Kraus VB, Birmingham J, Stabler TV, Feng S, Taylor DC, Moorman CT, Garrett WE, Toth AP (2012). Effects of intraarticular IL1-Ra for acute anterior cruciate ligament knee injury: a randomized controlled pilot trial (NCT00332254). Osteoarthritis Cartilage.

[46] Neidhart M, Gay RE, Gay S (2000). Anti–interleukin-1 and anti-CD44 interventions producing significant inhibition of cartilage destruction in an in vitro model of cartilage invasion by rheumatoid arthritis synovial fibroblasts. Arthritis Rheum.

[47] Frisbie DD, Kawcak CE, Werpy NM, Park RD, McIlwraith CW (2007). Clinical, biochemical, and histologic effects of intra-articular administration of autologous conditioned serum in horses with experimentally induced osteoarthritis. Am J Vet Res.

[48] Hyc A, Osiecka-Iwan A, Niderla-Bielinska J, Moskalewski S (2011). Influence of LPS, TNF, TGF-β1 and IL-4 on the expression of MMPs, TIMPs and selected cytokines in rat synovial membranes incubated in vitro. Int J Mol Med.

[49] Oguchi T, Ishiguro N (2004). Differential stimulation of three forms of hyaluronan synthase by TGF-β, IL-1β, and TNF-α. Connect Tissue Res.

[50] Alaaeddine N, Di Battista JA, Pelletier J-P, Kiansa K, Cloutier J-M, Martel-Pelletier J (1999). Inhibition of tumor necrosis factor α–induced prostaglandin E2 production by the antiinflammatory cytokines interleukin-4, interleukin-10, and interleukin-13 in osteoarthritic synovial fibroblasts: distinct targeting in the signaling pathways. Arthritis Rheum.

[51] Becker T, Tohidast-Akrad M, Humpeler S, Gerlag DM, Kiener H-P, Zenz P, Steiner G, Ekmekcioglu C (2014). Clock gene expression in different synovial cells of patients with rheumatoid arthritis and osteoarthritis. Acta Histochem.

[52] Weibrich G, Hansen T, Kleis W, Buch R, Hitzler WE (2004). Effect of platelet concentration in platelet-rich plasma on peri-implant bone regeneration. Bone.

[53] Fortier LA, Cole BJ (2014). Anti-inflammatory and matrix restorative mechanisms of platelet-rich plasma in osteoarthritis: response to Andia and Maffulli. Am J Sports Med.

